# Atlas of Tumor and Tumor Microenvironment Cells of Lymphovascular Space Invasion (LVSI) in High-Grade Serous Endometrial Adenocarcinoma: A Case Study

**DOI:** 10.3390/ijms25063441

**Published:** 2024-03-19

**Authors:** Raed Sulaiman, Adam Dale, Xiaoqian Lin, Jennifer C. Aske, Kris Gaster, David Starks, Luis Rojas Espaillat, Pradip De, Nandini Dey

**Affiliations:** 1 Department of Pathology, Avera Cancer Institute, Sioux Falls, SD 57108, USA; raed.sulaiman@plpath.org; 2 Translational Oncology Laboratory, Avera Cancer Institute, Sioux Falls, SD 57108, USApradip.de@avera.org (P.D.); 3 Assistant VP Outpatient Cancer Clinics, Avera Cancer Institute, Sioux Falls, SD 57108, USA; 4 Department of Gynecologic Oncology, Avera Cancer Institute, Sioux Falls, SD 57108, USA; 5 Department of Internal Medicine, University of South Dakota SSOM, Sioux Falls, SD 57108, USA; 6 Viecure, Greenwood Village, CO 80111, USA

**Keywords:** invasive HGS endometrial adenocarcinoma, LVI, tumor compartment, immune cells, epithelial cells, mesenchyme cells, cancer-associated fibroblast cells

## Abstract

Lymphovascular invasion (LVSI) is defined as the presence of tumor cells within a definite endothelial-lined space (lymphatics or blood vessels) in the organ surrounding invasive carcinoma. The presence of LVI is associated with an increased risk of lymph nodes and distant metastases. Lymphovascular invasion is described as cancer within blood or lymph vessels and is an independent risk factor for metastasis, recurrence, and mortality. This study aims to present the marker-based immunohistological characterization of cells around LVSI in a high-grade adenocarcinoma of the endometrium to build a cellular atlas of cells of LVSI. A cellular characterization of the cells around lymphovascular space invasion in a 67-year-old female patient with invasive high-grade serous endometrial adenocarcinomas is presented. Resected tumor tissue from a consented patient with invasive high-grade serous endometrial adenocarcinoma was obtained within an hour of surgery. The expressions of the epithelial markers (CK8, 18, and EpCAM), LCA (leukocyte common antigen) marker (CD45), proliferation marker (Ki67), apoptosis markers (cleaved PARP and cleaved caspase3), immune cell markers (CD3, CD4, CD8, CD56, CD68, CD163, FoxP3, PD-1, PD-L1), pro-inflammatory marker (IL-12-RB2), and fibroblast/mesenchyme markers (S100A7, SMA, and TE-7) of the resected tissue on the IHC stains were evaluated and scored by a pathologist. Acknowledging the deterministic role of LVSI in a high-grade adenocarcinoma of the endometrium, our study presents the first marker-based immunohistological atlas of the tumor and TME compartments in the context of epithelial cell markers, proliferation markers, apoptosis markers, macrophage markers, and fibroblast markers. Our study demonstrates that an aggressive disease like a high-grade adenocarcinoma of the endometrium inflicts the pro-metastatic event of LVSI by involving the immune landscape of both tumor and TME. This study demonstrates, for the first time, that the tumor cells within LVSI are positive for IL-12R-B2 and S100A4.

## 1. Introduction

Lymphovascular space invasion (LVSI), histological grade, and myometrial invasion are important prognostic factors of endometrial carcinoma, one of the most frequent gynecological cancers in developed countries [1]. LVSI, found in up to 35% of patients with endometrial cancers [2], is pathologically defined as the detection of tumoral cells in lymphatics or small vessels outside the core tumor. LVSI is characterized by the tumor cells within endothelium-lined spaces around the primary tumor that are observed as “free-floating” cell clusters, which frequently fit the space shape.

Uterine serous carcinoma is an uncommon form of endometrial cancer [3]. Serous carcinoma is the prototype of type-II endometrial cancer and accounts for 10% of all endometrial carcinomas; although rare, they are very aggressive tumors and are regarded as high-grade [4]. Despite its rarity, uterine serous carcinoma accounts for a disproportionate number of endometrial cancer deaths (estimated at approximately 40%; see [5]). The poor clinical outcomes for patients are attributable, in part, to an increased risk for occult metastases as compared to endometrioid endometrial carcinoma (See [4]).

The significance of LVSI in uterine serous carcinoma is recognized [6]. The presence and extent of LVSI and cervical stromal invasion are reported predictors for lymph node metastasis in uterine serous carcinoma [7].

In this case study, we have built a cellular atlas of LVSI in HGS-invasive endometrial adenocarcinoma. In this effort, we have used six types of marker-based characterization of the tumor compartment and cells of the tumor microenvironment (TME), including epithelial markers, LCA (leukocyte common antigen) markers, proliferation markers, apoptosis markers, immune cell markers, and fibroblast (mesenchyme) markers.

## 2. Patient Information and Consent

A 67-year-old female patient with invasive high-grade serous endometrial adenocarcinoma with no history of chemotherapies, undergoing surgical procedure of hysterectomy, BSO, and SLN dissection in 2021 consented to the ex vivo study as approved by the Avera IRB. The patient’s CA125 was 18.7 two weeks prior to the surgery.

### 2.1. Pathology

The final diagnosis was performed by a pathologist from the routine H and E (Hematoxylin and Eosin)-stained FFPE (Formalin-Fixed Paraffin-Embedded) sections from the resected tissues: hysterectomy, bilateral salpingo-oophorectomy, and sentinel and non-sentinel lymph node sampling and omentectomy. Invasive high-grade serous endometrial adenocarcinoma with deep myometrial invasion (87% of the uterine wall; depth of myometrial invasion was 19 mm of 22 mm uterine wall thickness) and lymphovascular space invasion was reported. There was a focal extension of carcinoma into the cervical stroma only; and the cervical and parametrial margins were free of malignancy. The serosa, fallopian tubes, and ovaries were intact and univloved. The sentinel lymph nodes (right external iliac, one lymph node; left obturator, two lymph nodes; and left pelvic, two lymph nodes) were free of malignancy. The periaortic non-sentinel lymph nodes showed micrometastatic carcinoma involving one of three lymph nodes. Very focal lymphovascular invasion is seen in the form of small clusters of tumor cells within delicate vascular spaced with identifiable endothelial cells recognized on routine H and E-stained sections. With these findings, the tumor was pathologically staged as pT2 p(sn)N2mi (AJCC 8th Edition).

### 2.2. Genomics

Serous uterine carcinomas are a limited feature of any known hereditary cancer syndrome [4]. This study evaluated the familial risk of cancers for patients with serous uterine carcinoma, focusing on Lynch syndrome malignancies, and identified a significant excess of ovarian and endometrial cancers in relatives of patients with endometrial cancer with pure serous and mixed serous tumors based on detailed three-generation family history data and medical record confirmation of malignancies in a single-institution cohort [4]. An IHC (ImmunoHistochemistry) ancillary test for MMR (Mis-Matched Repair) was performed. Ventana monoclonal antibodies against MSH2 (clone G219-1129), PMS2 (clone A 16-4), MLH 1 (clone M 1), and MSH6 (clone SP93) were evaluated. MLH1, MSH2, MSH6, and PMS2 were normal (intact nuclear expression), indicating an MMR-proficient tumor. BRCA germline mutations in women with uterine serous carcinoma are a topic of debate [8]. BRCA 1/2 Analyses with CustoinNext-Cancer^®^ +RNA/nsight^®^ showed no pathogenic mutations, variants of unknown significance, gross deletions or duplications, and no clinically relevant aberrant RNA transcripts.

### 2.3. Tissue Collection at the Time of Surgery

All experimental protocols were approved by the institutional and/or licensing committee(s). Informed consent (IRB approved: Protocol Number Study: 2017.053-100399_ExVivo001) was obtained from the patient. The resected tumor tissue from the patient was collected during a surgical procedure (total hysterectomy and bilateral salpingo-oophorectomy) in designated collection media as per the guidelines and relevant regulations provided by the pathologist.

### 2.4. Immunohistological Marker-Based Characterization of Tumor and TME Compartments

Tumor cells and cells of TME around LVSI were characterized using immunological markers, including epithelial markers, LCA markers, proliferation markers, apoptosis markers, immune cell markers, and fibroblast (mesenchyme) markers. Morphological and immunohistological staining of the FFPE sections from the resected tumor tissue was performed, as mentioned earlier [9]. In short, IHC expression of PD-L1(22C3; Dako # M3653) and PD-1 (ABCAM # ab137132) was carried out on FFPE sections from the tumor by double stain. All IHC expression of PD-L1 and PD-1 was performed on resected tissues, which were processed within an hour of surgery to preserve different types of cells, including tumor cells and cells of the TME. Tumor samples were tested for the IHC expression of CD3 (Anti-CD3 [SP7] ab16669), CD4 ([EPR6855] ab133616), CD8 (ab85792), CD68 (Dako. #M0876), CD163 (Cell Signaling #93498), FoxP3 ([236A/E7] ab20034) in immune cells and PD-L1 (Clone 22C3; Dako. #M3653) in tumor cells and tumor-associated macrophages. The IHC expression of EpCAM, CD45, CK 8, 18, S100A4, SMA, TE-7, Ki-67, cleaved-PARP, cleaved-caspase3, CD3, CD4, CD8, CD56, CD68, CD163, IL12R-B2, and FoxP3 and PD-L1 from the tumor was carried out on FFPE sections. The IHC detection kits were procured from Dako (Envision+ Dual-link system-HRP (DAB+), code K4065; Envision GI2 Doublestain system, Rabbit/Mouse (DAB+/Permanent Red), code K5361), and Abcam (ab210059 DoubleStain IHC Kit: M&R on human tissue (DAB and AP/Red). The validation of the protein expression was carried out in FFPEs of tonsil and tumor tissues. A board-certified pathologist evaluated the staining intensity and distribution pattern of expression of proteins by applying the standard scoring protocol and guidelines using a standard scoring system. Table 1 presents the list of markers included in the case study.

### 2.5. Expression of Immuno-Histological Markers in Tumor Cells and Cells of TME Compartments

**Figure 1:**
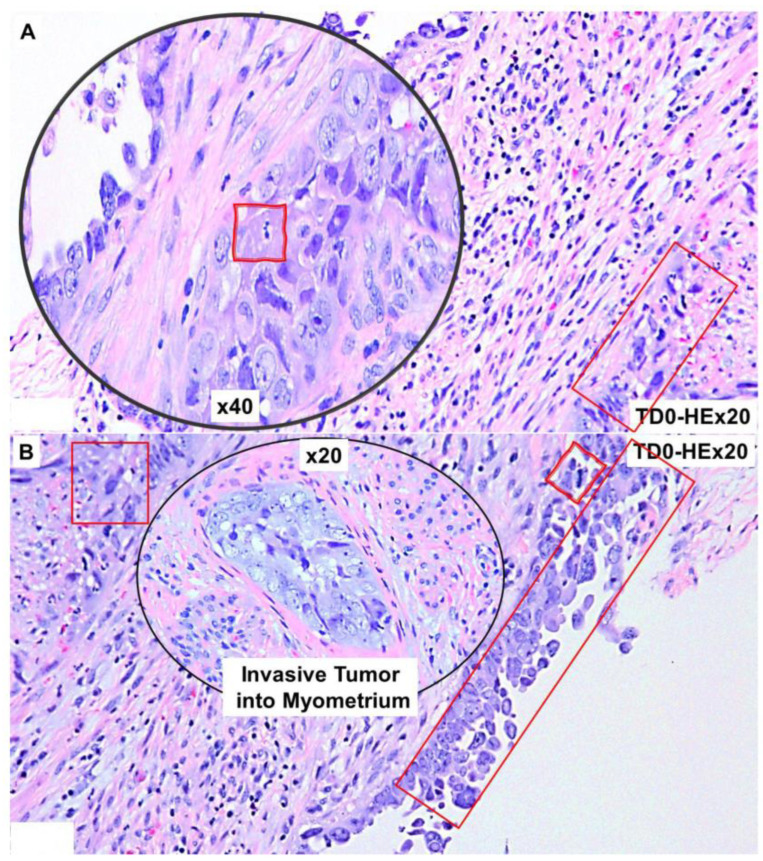

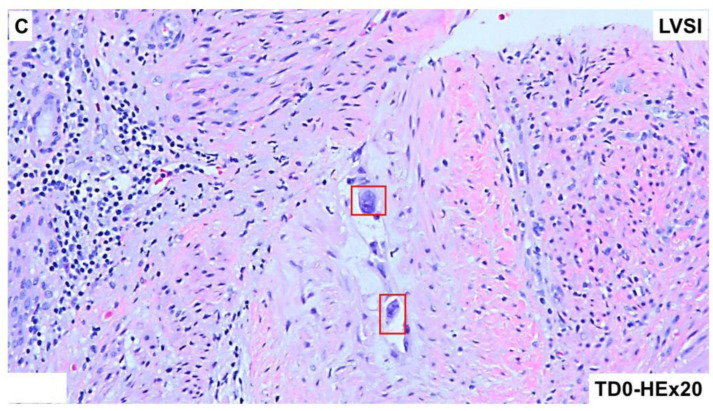
Microscopic features of the high-grade invasive uterine serous carcinoma with LVSI: H&E-stained FFPE section of the resected tumor tissue shows microscopic characteristics of uterine serous carcinomas. Marked nuclear atypia and solid growth with mitotic bodies are observed (**A**). The invasive nature of the tumor cells in the myometrium with mitotic figures (**B**) and tumor cells within blood vessels are seen as a typical sign of LVSI along with high inflammatory reactive TILs (**C**). Magnifications are denoted in the photomicrographs. Tumor cells are presented in red squares. Tumor cells with mitotic figures are represented in red squares with scribbled double lines.

**Figure 2:**
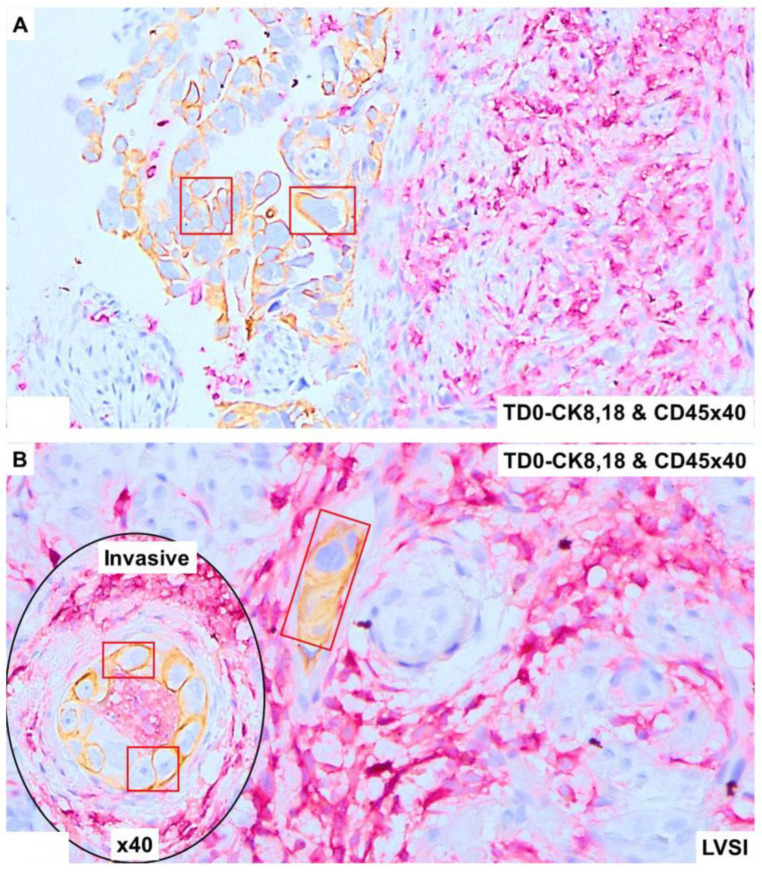

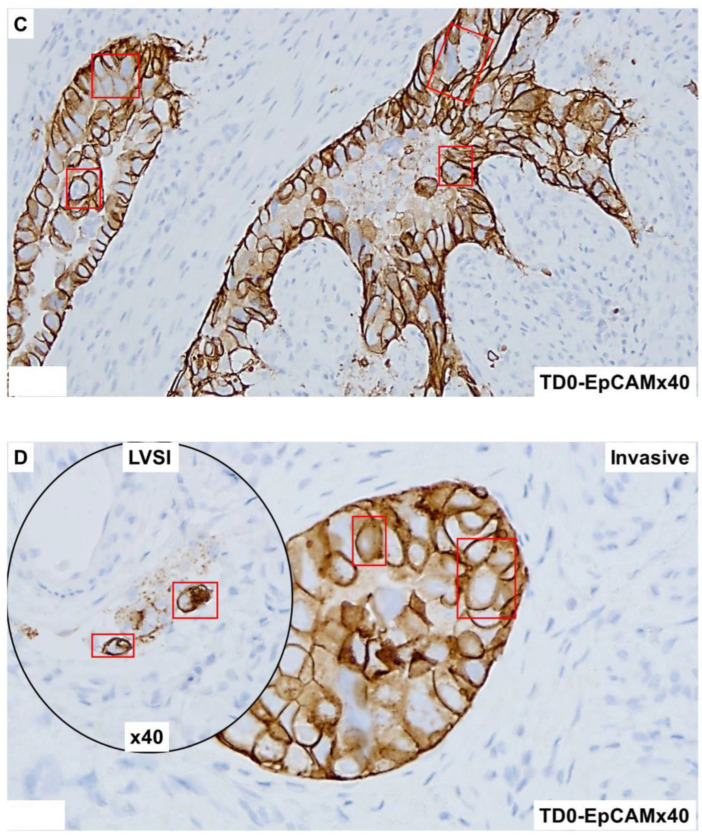
Expression of epithelial cell markers and LCA markers in the high-grade invasive uterine serous carcinoma with LVSI: Microscopic features of the tumor compartment and TME was revealed by the double IHC stain for CK8,18 and CD45 (**A**,**B**) and EpCAM (**C**,**D**). The epithelial tumor cells on the surface of the tumor were positive (brown DAB color) for the epithelial marker CK8,18 and negative for the LCA marker CD45. Nucleii showed prominent atypia (hematoxylin as counter stain) with scant cytoplasm in contrast to the highly positive (pink alkaline phosphatase color) TILs in TME (**A**). CK8, 18 positive, and CD45-negative tumor cells were observed in the invasive tumor and within the characteristic LVSI (**B**). Membranous EpCAM positivity of tumor cells is seen in the invasive tumor regions (**C**) and LVSI regions (**D**). Magnifications are denoted in the photomicrographs. Tumor cells are presented in red squares.

Morphologically, the tumor demonstrated marked nuclear atypia, solid growth, and mitotic bodies. Figure 1 presents the LVSI in the resected tumor sample showing characteristic histopathological features of high-grade serous adenocarcinoma of endometrium by H&E. The invasive nature of the histology (Figure 1A) and the invasive tumor into the myometrium (Figure 1B) is observed along with a representative LVSI (Figure 1C). We tested the epithelial and leucocyte common antigen (LCA) marker expressions in cells around LVSI in the resected tumor sample, as seen in Figure 2.

**Figure 3:**
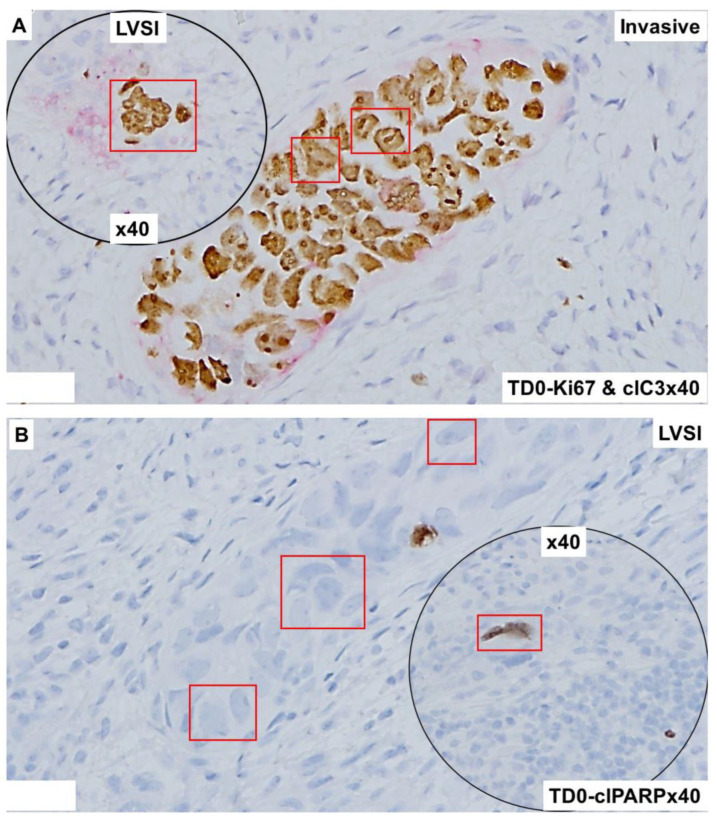
Expression of proliferation and apoptotic markers in the high-grade invasive uterine serous carcinoma with LVSI: Microscopic features of the tumor compartment and TME was revealed by the double IHC stain for Ki67 and cleaved caspase3 (**A**) and cleaved PARP (**B**). The epithelial tumor cells of the invasive part of the tumor were positive (brown DAB color) for the proliferation marker Ki67 and negative for the apoptotic marker cleaved caspase3 (pink alkaline phosphatase color). Nucleii showed prominent atypia in the Ki67 stain with scant cytoplasm. Ki67 positive and cleaved caspase3 negative tumor cells were observed in the invasive tumor and within the characteristic LVSI (**A**). Likewise, a scant cleaved PARP stain was observed in tumor cells in the region of LVSI (**B**). Magnifications are denoted in the photomicrographs. Tumor cells are presented in red squares.

**Figure 4:**
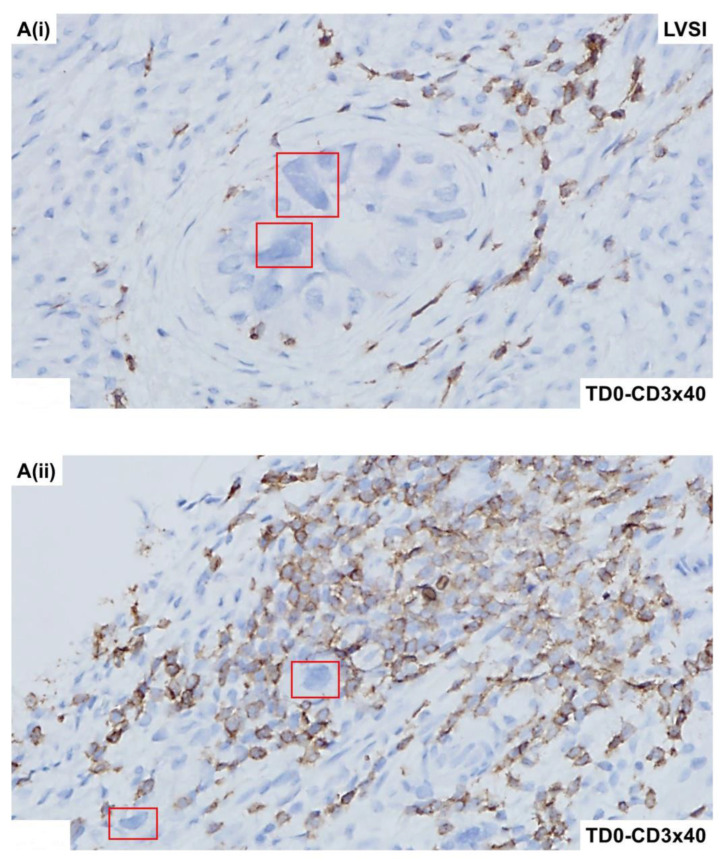

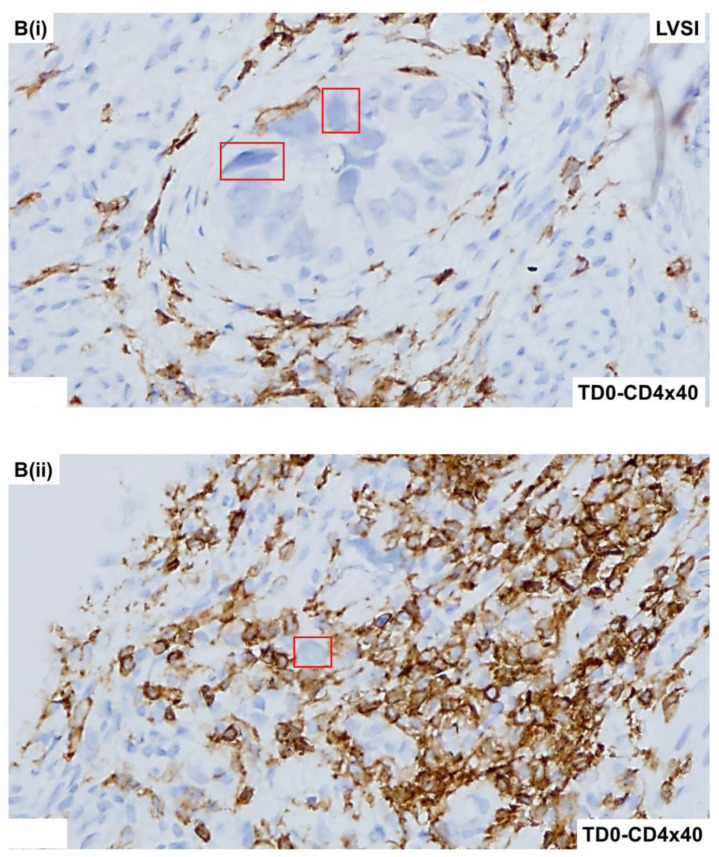

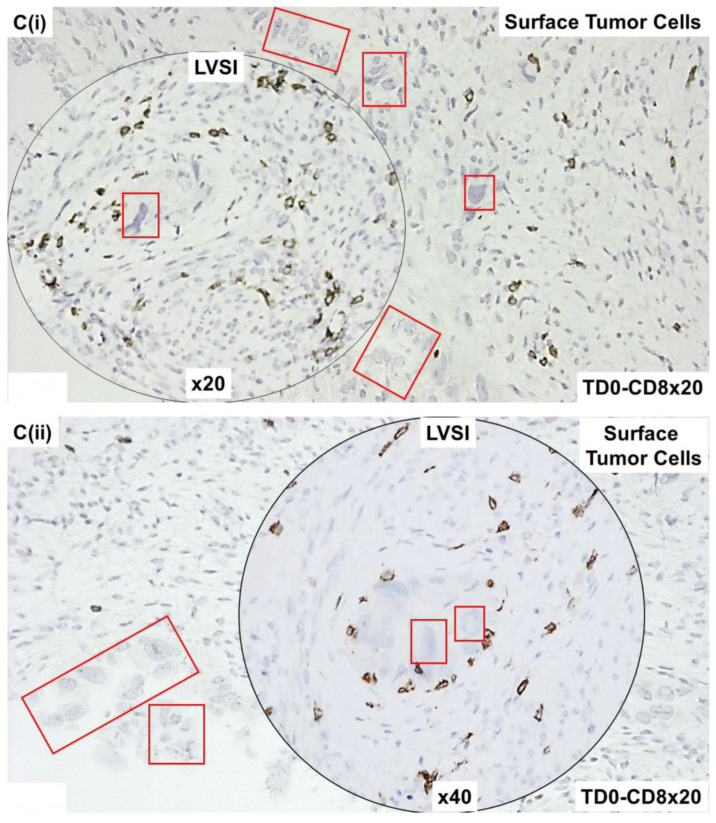

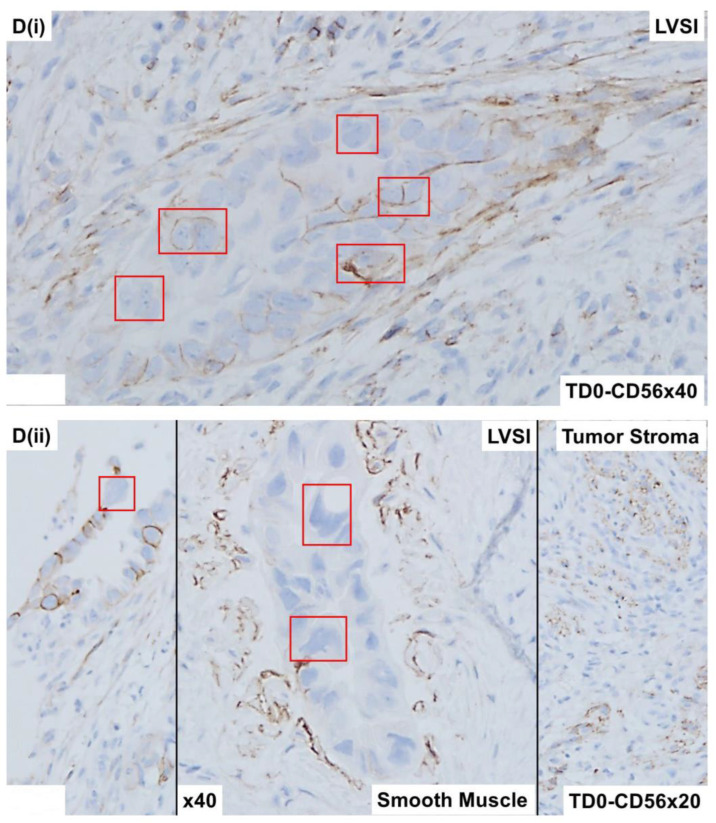

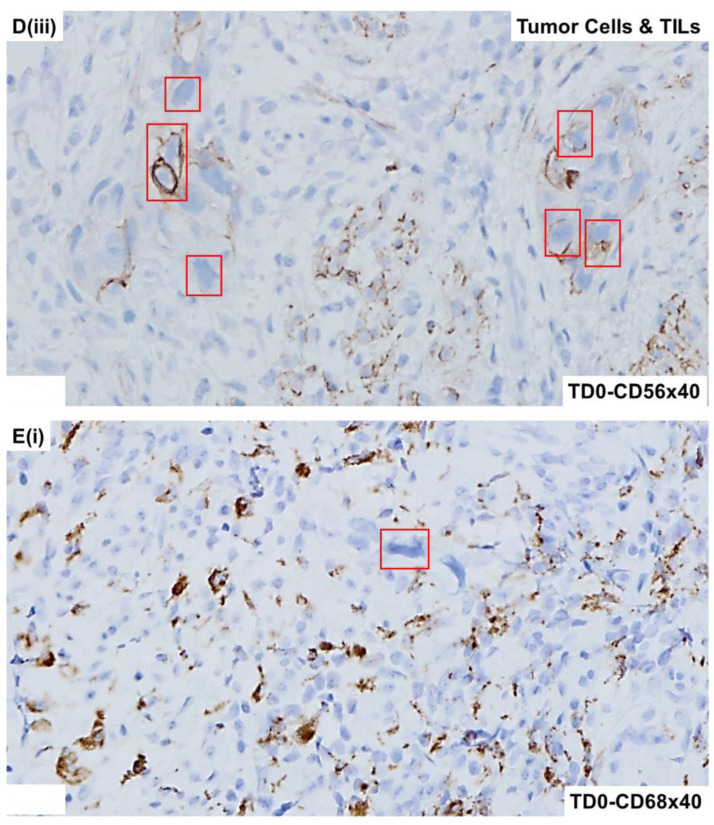

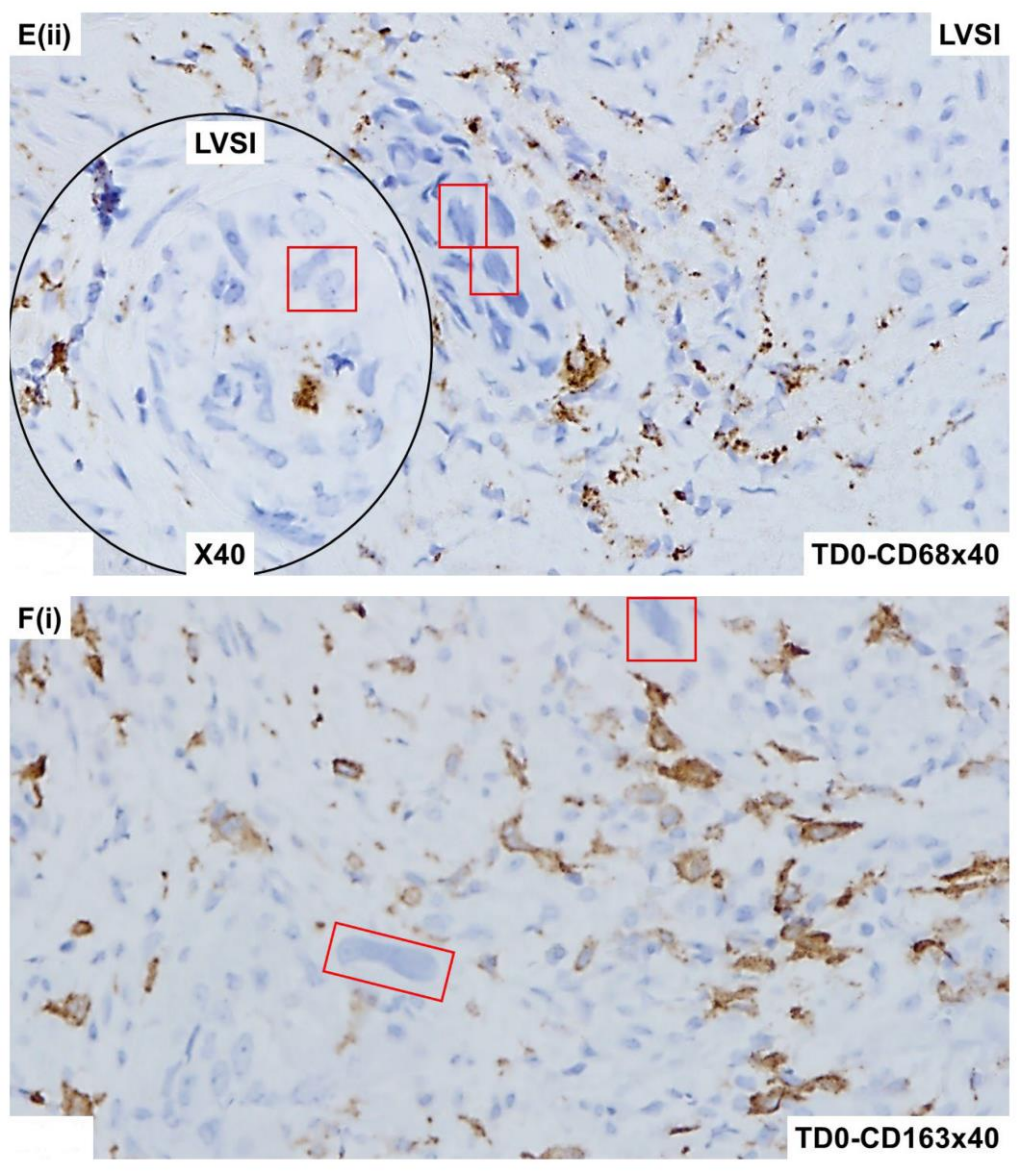

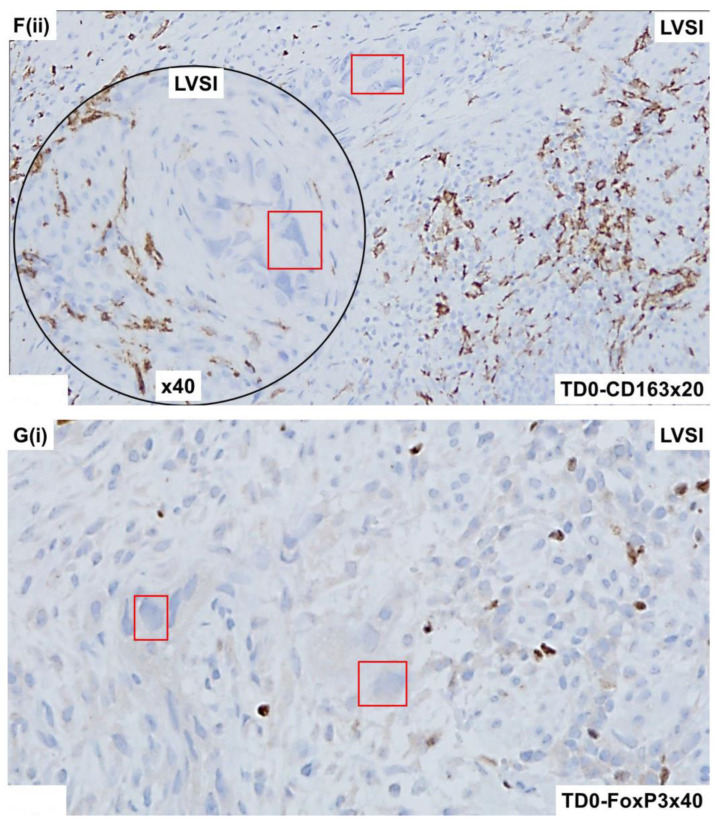

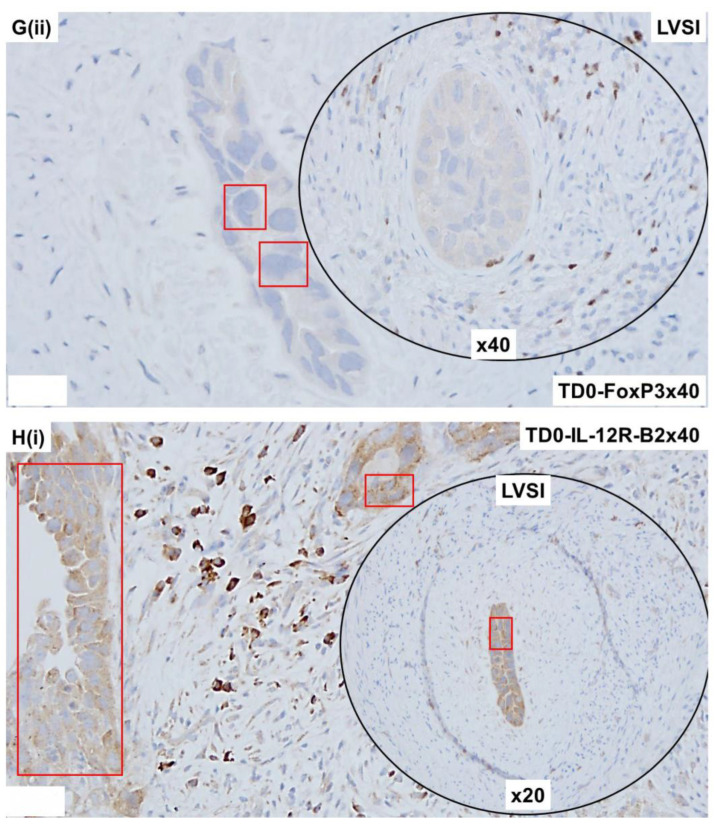

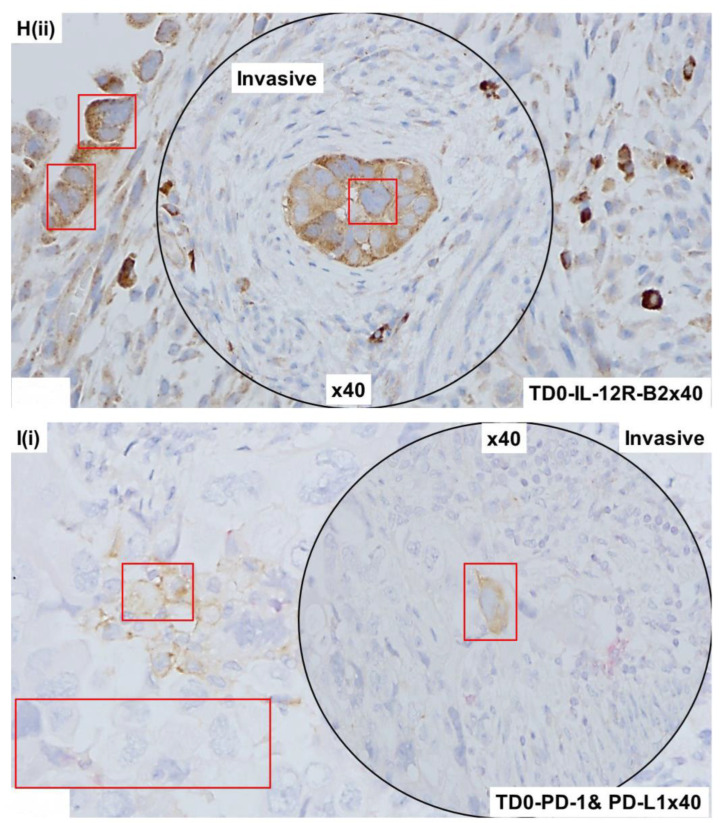

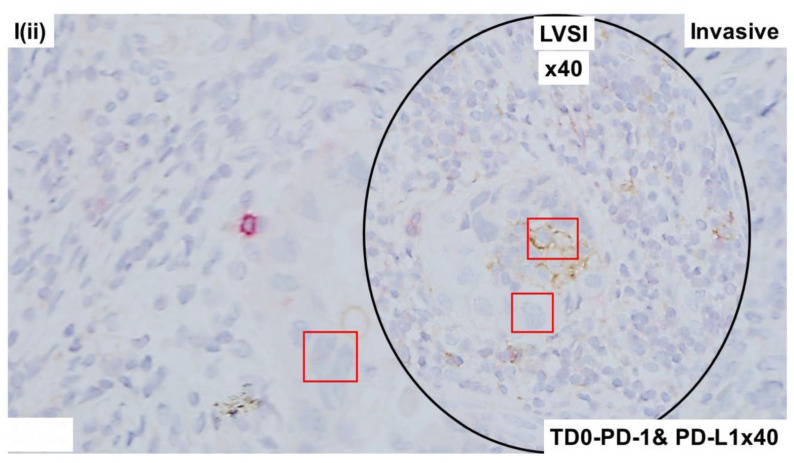
Expression of immune cell markers and immune markers in the high-grade invasive uterine serous carcinoma with LVSI: Microscopic features of the tumor compartment and TME were revealed by the IHC stain for CD3 **A**(**i**,**ii**), CD4 **B**(**i**,**ii**), CD8 **C**(**i**,**ii**), CD56 **D**(**i**–**iii**), CD68 **E**(**i**,**ii**), CD163 **F**(**i**,**ii**), FoxP3 **G**(**i**,**ii**), IL-12R-B2 **H**(**i**,**ii**), and PD-1-PD-L1 double stains **I**(**i**,**ii**). Both CD3 and CD4-positive TILs were more in number on the surface tumor region compared to the LVSI regions. In contrast, the expression of CD8 was lesser in both the tumor surface and the LVSI region. The expression of CD56 was found in some tumor cells and TILs in both the regions of LVSI and tumor stroma. The expression of the marker varied qualitatively (incomplete or complete) and quantitatively (rare zonal positive or highly positive) in tumor cells. On the contrary, as expected, macrophage marker expression was uniformly absent in tumor cells. The expression of CD63 and CD163 was found to be granular. The expression of FoxP3 was limited only to the TILs and quantitatively was rare in the tumor tissue. The granular cytoplasmic expression of IL-12-B2 was found in tumor cells uniformly in both regions of invasion and LVSI. However, the degree of expression was much lesser than that of the highly positive TILs. The tumor cells and TILs of the invasive and LVSI region expressed PD-L1 (brown DAB color), while minimal expression of PD-1 (pink alkaline phosphatase color) was present on TILs. Tumor cells were negative for all T-cell and macrophage markers (CD3, CD4, CD8, CD68, CD163, FoxP3, and PD-1). Magnifications are denoted in the photomicrographs. Tumor cells are presented in red squares.

**Figure 5:**
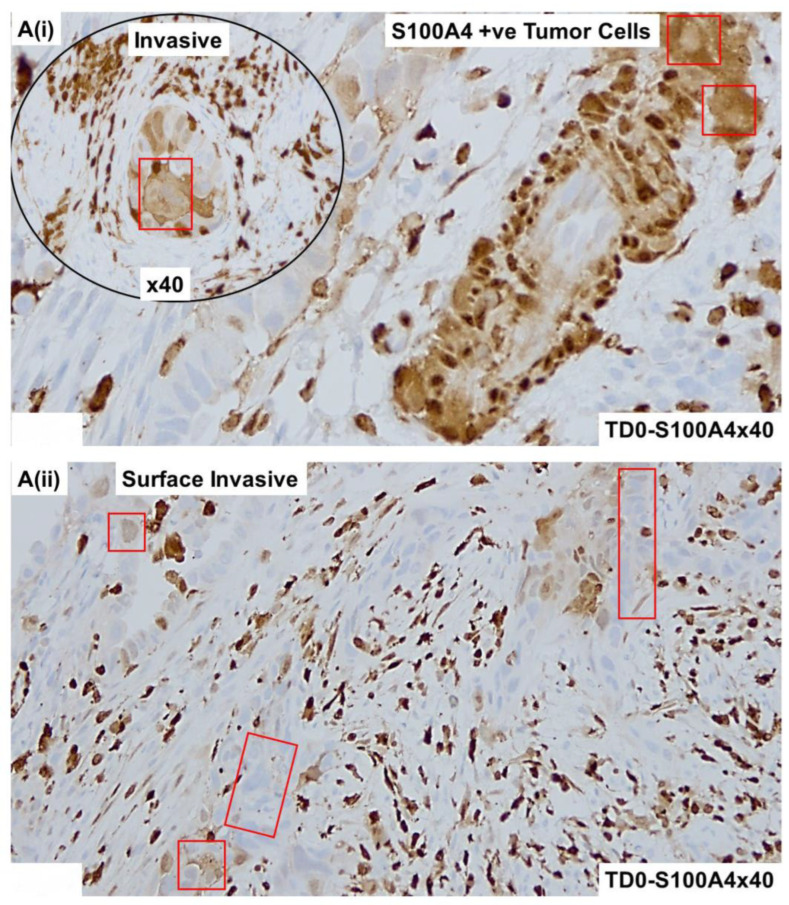

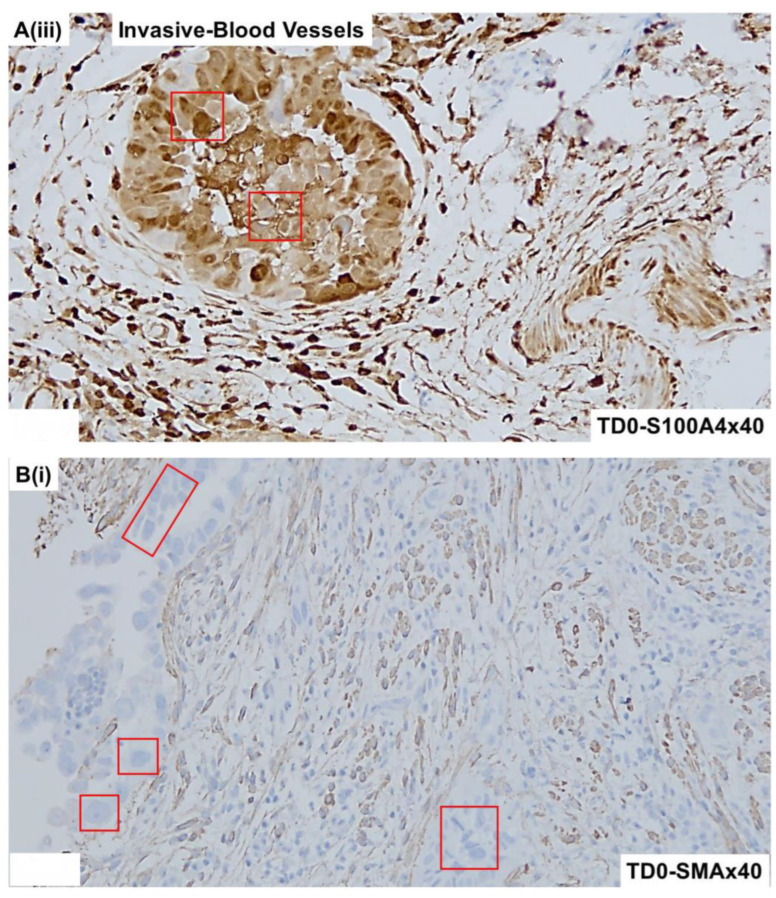

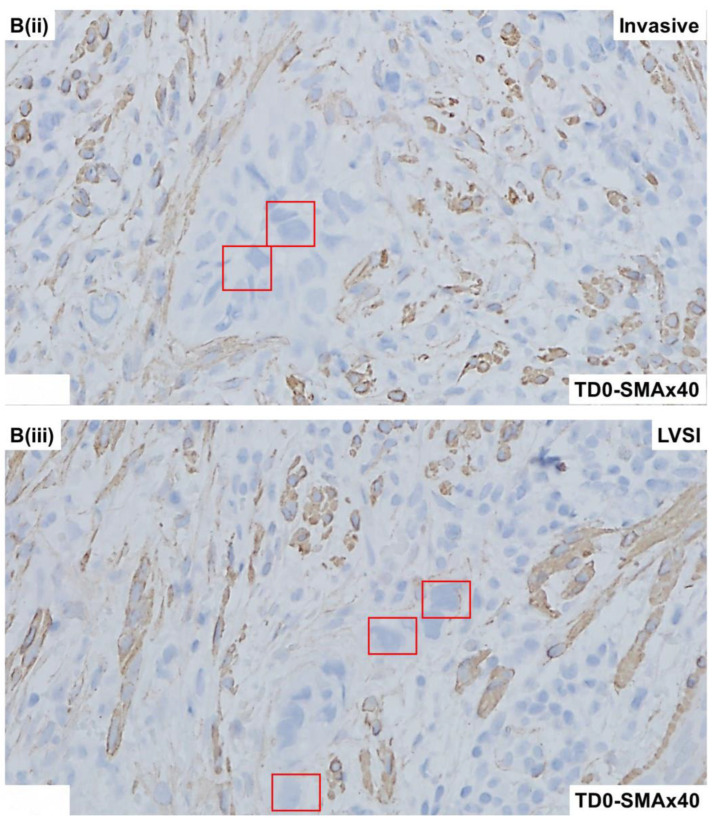

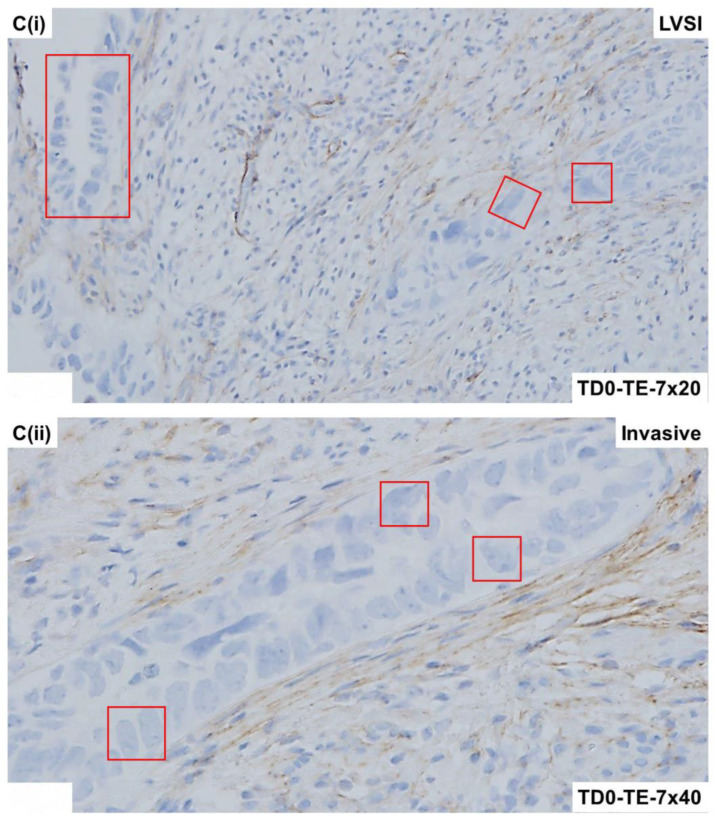

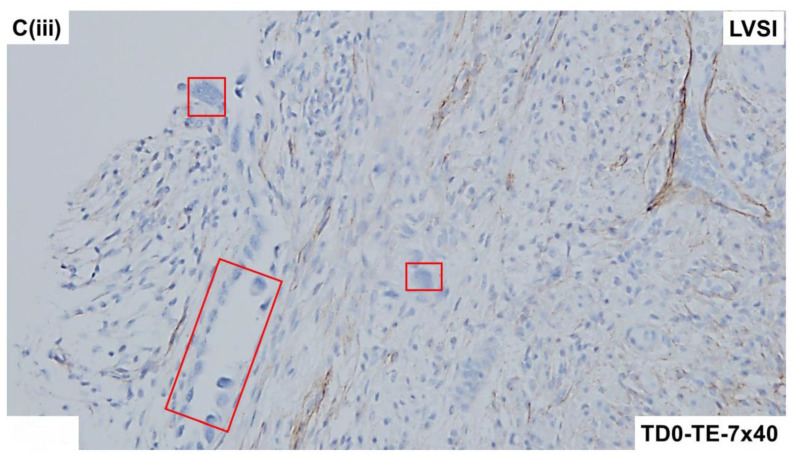
Expression of mesenchymal cell markers and fibroblast markers in the high-grade invasive uterine serous carcinoma with LVSI: Microscopic features of the tumor compartment and TME were revealed by the IHC stain for S100A4 **A**(**i**–**iii**), SMA **B**(**i**–**iii**), and fibroblast marker TE-7 **C**(**i**–**iii**). In addition to the mesenchymal positivity of S100A4 in the TME, we observed moderate-to-high positive stains for S100A4 in tumor cells in both the invasive and regions of LVSI. Both SMA and TE-7 were expressed in mesenchyme cells and were negative for the tumor cells in both invasive and around LVSI. Magnifications are denoted in the photomicrographs. Tumor cells are presented in red squares.

The invasive nature of the histology of the tumor cells positive for CK8,18 and negative for CD45 as double stained with CK8,18 and CD45 is presented (Figure 2A,B). Epithelial tumor cells were negative for CD45 (alkaline phosphatase stain in pink) and positive for CK 8,18 (DAB stain in brown). The invasive tumor cells of the LVSI (Figure 2C,D) with membranous EpCAM positivity are presented. We stained the tumor sample with proliferation and apoptosis markers around LVSI, as shown in Figure 3. Epithelial tumor cells were found to be mostly negative for apoptosis markers, cleaved caspase3 (alkaline phosphatase stain in pink), and cleaved PARP (DAB stain in brown), while strongly nuclear positive for the proliferation marker, Ki67 (DAB stain in brown). The invasive tumor cells were found to be positive for Ki67 with rare positivity for cleaved caspase3 as stained simultaneously with Ki67 and cleaved caspase3 double stain (Figure 3A). In contrast, the invasive tumor cells of the LVSI (Figure 2B) were rarely stained with cleaved PARP. Figure 4 shows the immune cell marker expressions around the LVSI. The invasive nature of the histology of the non-tumor cells and tumor cells of the LVSI were demonstrated with CD3 (Figure 4A(i,ii)), CD4 (Figure 4B(i,ii)), CD8 (Figure 4C(i,ii)), CD56 (Figure 4D(i–iii)), CD68 (Figure 4E(i,ii)), CD163 (Figure 4F(i,ii)), FoxP3 (Figure 4G(i,ii)), IL-12RB2 (Figure 4H(i,ii)), and PD-1 and PD-L1 double stain (Figure 4I(i,ii)). Epithelial tumor cells were found negative for TIL markers, macrophage markers, and PD-1. Tumor cells of LVSI were positive for the pro-inflammatory marker, IL-12RB2. TILs were positive for PD-1 (alkaline phosphatase stain in pink) and PD-L1 (DAB stain in brown) in double stain. Immune cell marker expressions in the T-cells were membranous. Finally, the fibroblastic and tumor mesenchyme marker expressions demonstrated the fibroblastic nature of the TME mesenchyme in cells around LVSI (Figure 5). The invasive nature of the histology and the invasive tumor cells of the LVSI were stained with S100A4 Figure 5A(i–iii), SMA Figure 5B(ii,iii), and TE-7 Figure 5C(i–iii) single IHC stains (DAB stain in brown). Epithelial tumor cells were found to be mostly positive for S100A4, although the intensity of the stain varied between tumor cells. In contrast, both SMA and TE-7 were expressed in mesenchyme cells. The tumor cells were negative for SMA and TE-7.

## 3. Discussion

As LVSI has been recognized as one of the initial events in the lymphomatous and hematogenous metastases in endometrial carcinoma [3], LVSI is associated with lower overall survival, higher risk of recurrence, lymph node metastasis, and distant metastasis that LVSI correlates with poorer prognosis in endometrial cancers of FIGO stage I–III. The LVSI is associated with higher histologic grade and deep MMI and is considered an independent poor prognostic factor in endometrial carcinoma [10]. We observed no histomorphologic differences in the LVSI features among endometrial adenocarcinoma subtypes. Recently, LVSI in endometrial carcinoma has been known to be a prognostic factor independent of molecular signature [11]. The deep myometrial invasion correlates to positive LVSI, positive LNM, cancer recurrence, and poor OS for endometrial cancer patients [12]. Due to the high frequency of hematogenous dissemination in patients with serous carcinoma, LVSI is the primary feature of the dissemination. Here, we studied the cellular characterization of LVSI in a case of high-grade (3) and stage (IIIC2) invasive serous adenocarcinoma patients. Since we studied the LVSI, we also tested the patient’s blood for circulating tumor cells (CTCs) and circulating cancer-associated macrophage-like cells (CAMLs) on the day of the surgery. Although IHC can aid in the identification of LVSI in difficult or challenging cases, keeping in mind the histopathological features of our case, in line with other reports [13,14], we used standard H&E to aid in the diagnosis of LVSI in our case. As mentioned earlier, CTC and CAMLs were identified in our laboratory [15,16]. The patient’s blood was positive for both CTC and CAMLs.

Our study demonstrates, for the first time, that the tumor cells within LVSI were positive for IL-12R-B2 and S100A4. As an ideal example of cancer associated with epithelial–mesenchymal transition (EMT), which exhibits cancer stem cell (CSC)-like traits, uterine carcinosarcoma, more specifically, a high-grade form exhibiting LVSI, demands an in-depth understanding of the expression of EMT markers, like S100A4. S100A4 is a well-established tumor metastasis mediator in endometrial cancers, and estrogen-related receptor γ knockdown transcriptionally inhibits S100A4 expression to promote the expression of its downstream target E-cadherin, and vice versa [17]. Although S100A4 is a known inducer of EMT in uterine tumorigenesis [18], little is known about its involvement in LVSI and its differentiation. In a cell line-based study, Tochimoto et al. reported that cell lines stably overexpressing S100A4 enhanced CSC properties and decreased cell proliferation and acceleration of cell migration [19]. In their clinical samples, the S100A4 score was found to be significantly higher in sarcomatous as compared with carcinomatous components and was positively correlated with ALDH1, Slug, and vimentin scores and inversely with Ki-67-labeling indices. The study suggested the involvement of a S100A4-related signaling cascade, although initiated in the carcinomatous tumor components, to the establishment of EMT properties but can be a part of the event toward a divergent sarcomatous differentiation. The presence of LVSI indicates that the tumor is histopathologically predisposed to metastatic progression. S100A4 is known for its role in a number of cellular processes, including cell-cycle progression and metastasis. Hence, it is logical to argue that the S100A4 positivity of tumor cells in the LVSI is associated with poor prognosis. Our study demonstrates, for the first time, that the tumor cells within LVSI were positive for S100A4, highlighting its role as a promising prognostic marker in endometrial adenocarcinoma, especially in high-grade LVSI form.

The endometrial cancer (EC) cells in the LVSI in our study appeared to be positive with S100A4 IHC signal. S100A4 has been thought of as a crucial EMT mediator since the Wnt/B-catenin signaling pathway directly controls it, more specifically in the context of addressing the activation of the Wnt/B-catenin pathway in a diverse landscape of endometrial carcinogenesis [20]. Studies have reported that S100A4 promotes endometrial cancer progress through epithelial–mesenchymal transition regulation [18]. S100A4 promoted the migration and invasion capacity of EC cells via EMT modification. In their study, Hua et al. reported that an aberrant S100A4 expression may predict EC progression and play an important role in regulating EC cell invasion through EMT regulation, indicating that S100A4 is a promising therapeutic target. Although a future confirmation is warranted, it is very likely that the tumor cells are undergoing EMT.

When a tumor undergoes EMT, it is presumed that the intrinsic tumor-suppressor mechanisms have failed, and hence, it is understood that the CSCs experience extrinsic pressure from tumor-infiltrating lymphocytes (TILs) [21,22]. As various TILs interact with secreted cytokines in the tumor microenvironment and typically either kill tumors at early stages or promote tumors at advanced stages, CSCs and TILs form the “three-E” sequential cancer immunoediting phases: elimination, equilibrium, and escape [21,22,23,24]. Although intrinsic mechanisms of epigenetic immunoediting for IL-12-RB2 are not clear, IL-12-RB2 regulates both the number and functional maturity of regulatory T cells. IL-12RB2 is a part of homeostasis within the tumor cells and TILs, and this homeostasis affects prognosis [25]. Our results show that the granular cytoplasmic expression of IL-12-B2 was found in tumor cells uniformly in both regions of invasion and LVSI. However, the degree of expression was much lesser than that of the highly positive TILs. Pan-cancer data also show a limited expression of IL-12-RB2 in endometrial tumor cells as compared to other solid tumors, namely melanoma, breast, kidney, and colorectal cancers. Our data warrant further studies to understand the prognostic significance of IL-12-B2 expression in endometrial cancers.

## 4. Conclusions

Our case study demonstrates the characteristics of tumor cells and cells of TME around LVSI in a rare yet aggressive form of endometrial adenocarcinoma. Epithelial tumor cells of an aggressive disease enforce several pro-metastatic event(s), including LVSI, via direct and indirect crosstalk with the immune and fibroblastic/mesenchymal stromal cells of TME. This study demonstrates, for the first time, that the tumor cells within LVSI are positive for IL-12R-B2 and S100A4.

## Figures and Tables

**Table 1:** List of markers included in the case study.

Compartments	IHC Stains *			
	Tumor Compartment		TME Compartment	
**Epithelial Cell Markers**	CK 8, 18			
	EpCAM			
**LCA Marker**			CD45	
**Proliferation Marker**		Ki67		
**Apoptosis Markers**		Cleaved-Caspase3		
		Cleaved-PARP		
**Immune-Cell Markers**			CD3	
			CD4	
			CD8	
			CD65 (NCAM1)	
			FoxP2	
			PD-1	
			IL-12R-B2	
**Immune Marker**			PD-L1	
**Macrophage** **Markers**			CD68	
			CD163	
**Fibroblast Markers**				S100A4
				SMA
				TE-7

* Color of the chromogens in the IHC stains of the specific proteins. (Brown for DAB and Pink for Alkaline phosphatase).

## Data Availability

This study was translational/experimental in nature. No public data were used. All experimental data are presented in the figures.
